# New diagnostic approach of the different types of isolated craniosynostosis

**DOI:** 10.1007/s00431-020-03860-9

**Published:** 2020-11-05

**Authors:** Sophia A. J. Kronig, Otto D. M. Kronig, Henri A. Vrooman, Jifke F. Veenland, Léon N. A. Van Adrichem

**Affiliations:** 1 Department of Plastic and Reconstructive Surgery and Hand Surgery, Dutch Craniofacial Centre, Erasmus MC - Sophia Children’s Hospital, University Medical Centre Rotterdam, Rotterdam, The Netherlands; 2 Department of Plastic and Reconstructive Surgery and Hand Surgery, University Medical Centre Utrecht, Heidelberglaan 100, 3584 CX Utrecht, The Netherlands; 3 Department of Radiology, Erasmus MC, Rotterdam, University Medical Centre Rotterdam, Rotterdam, The Netherlands; 4 Department of Medical Informatics, Erasmus MC, Rotterdam, University Medical Centre Rotterdam, Rotterdam, The Netherlands; 5 Department of Plastic and Reconstructive Surgery, University Medical Centre Utrecht, Utrecht, The Netherlands

**Keywords:** Craniosynostosis, Shape analysis, Decision-making, Computer-assisted diagnosis, Computed tomography

## Abstract

In this study, we diagnose skull shape deformities by analysing sinusoid curves obtained from standardized computed tomography (CT) slices of the skull for the common craniosynostoses (scaphocephaly, brachycephaly, trigonocephaly, right- and left-sided anterior plagiocephaly). Scaphocephaly has a high forehead peak and low troughs, in contrast to brachycephaly. Anterior plagiocephaly has asymmetry and shifting of the forehead peak. Trigonocephaly has a high and narrow frontal peak. Control patients have a symmetrical skull shape with low troughs and a high and broader frontal peak. Firstly, we included 5 children of every group of the common craniosynostoses and additionally 5 controls for extraction and calculation of characteristics. A diagnostic flowchart was developed. Secondly, we included a total of 51 craniosynostosis patients to validate the flowchart. All patients were correctly classified using the flowchart.

*Conclusion*: Our study proposes and implements a new diagnostic approach of craniosynostosis. We describe a diagnostic flowchart based on specific characteristics for every type of craniosynostosis related to the specific skull deformities and control patients. All variables are expressed in number; therefore, we are able to use these variables in future research to quantify the different types of craniosynostosis.

**Table:** 

**What is Known:** • *Premature fusion of one or more cranial sutures results in a specific cranial shape.* • *Clinical diagnosis is relatively simple; however, objective diagnosis based on distinctive values is difficult.*	
**What is New:** • *Using external landmarks and curve analysis, distinctive variables, and values for every type of craniosynostosis related to the specific skull deformities were determined and used to create a diagnostic flowchart for diagnosis.* • *Validation with an independent data set of 51 patients showed that all patients were correctly classified.*

**What is Known:**

• *Premature fusion of one or more cranial sutures results in a specific cranial shape.*

• *Clinical diagnosis is relatively simple; however, objective diagnosis based on distinctive values is difficult.*

**What is New:**

• *Using external landmarks and curve analysis, distinctive variables, and values for every type of craniosynostosis related to the specific skull deformities were determined and used to create a diagnostic flowchart for diagnosis.*

• *Validation with an independent data set of 51 patients showed that all patients were correctly classified.*

## Introduction

Craniosynostosis is the premature fusion of one or more of the cranial sutures. Four sutures are considered major: the metopic, coronal, sagittal, and lambdoid. Additionally, there are three minor sutures: the frontonasal, temporosquamosal, and frontosphenoidal. Premature fusion of one or more of the sutures results in characteristic anatomic malformations of the skull. The incidence of craniosynostosis is estimated to be 1 in 2000–2500 live births and may be either nonsyndromic (isolated) or syndromic [1].

Traditionally, the type of craniosynostosis is classified according to the synostotic suture(s) involved, the presence or absence of a skull-facial syndrome, and the (typical) shape of the cranial deformation. Scaphocephaly (sagittal synostosis) has a keel-like shape with an elongated anteroposterior and narrowed transverse dimension, whereas brachycephaly (bicoronal synostosis) has a reduced length with a retrusive forehead, orbits, and occiput and increased width [2, 3]. Trigonocephaly (metopic synostosis) has a characteristic triangular shape of the forehead and orbits, and anterior plagiocephaly (unilateral coronal synostosis (UCS)) is characterized by forehead and orbital asymmetry [2, 4, 5].

The diagnosis of craniosynostosis is primarily based on clinical examination. For confirmation or in case of diagnostic uncertainty, radiographic imaging is obtained. Currently, computed tomographic (CT) imaging is considered the standard for diagnosing craniosynostosis [6]. A descriptive classification can be made based on the affected suture.

In our previous study, we proposed a new method (UCSQ (Utrecht Cranial Shape Quantifier)) to classify skull shape deformities; from the resulting curves, specific values can be extracted and calculated [7]. In the present study, these variables, typical for different types of craniosynostosis, will be used to create a decisive and descriptive flowchart in establishing the diagnosis of skull shape deformities.

## Material and methods

### Patients

We included 25 children (age < 1 year) with nonsyndromic craniosynostosis for the development of the flowchart. Five children of every type of most common craniosynostosis (scaphocephaly, brachycephaly, trigonocephaly, and left- and right-sided UCS) were included. A pre-operative CT scan of the head needed to be available. In addition, 5 control patients were included. For the control data set, the CT scan needed to be made at an age of 6 years or younger. Children with other congenital or traumatic craniofacial malformations, including craniosynostosis of multiple sutures, facial fractures, or soft tissue swelling, were excluded. To be eligible as a control patient, the CT scan needed to contain the orbits and ears. These patients were also included in our previous study [7].

For validation of the aforementioned diagnostic flowchart, we additionally included patients of the following subgroups: scaphocephaly, brachycephaly, trigonocephaly, and left- and right-sided UCS. Inclusion criteria for these patients were the same as the first group.

All patients were diagnosed at the Erasmus Medical Centre, Sophia Children’s Hospital Rotterdam. The Erasmus MC Sophia Children’s Hospital is a specialized centre for the treatment of skull deformities.

The study was approved by the local Medical Ethics Review Committee (MEC-2016-467). The study was deemed a retrospective clinical study and did not require formal research ethics approval under the Medical Research Involving Human Subjects Act (WMO).

### Creating sinusoid curves

**Fig. 1:**
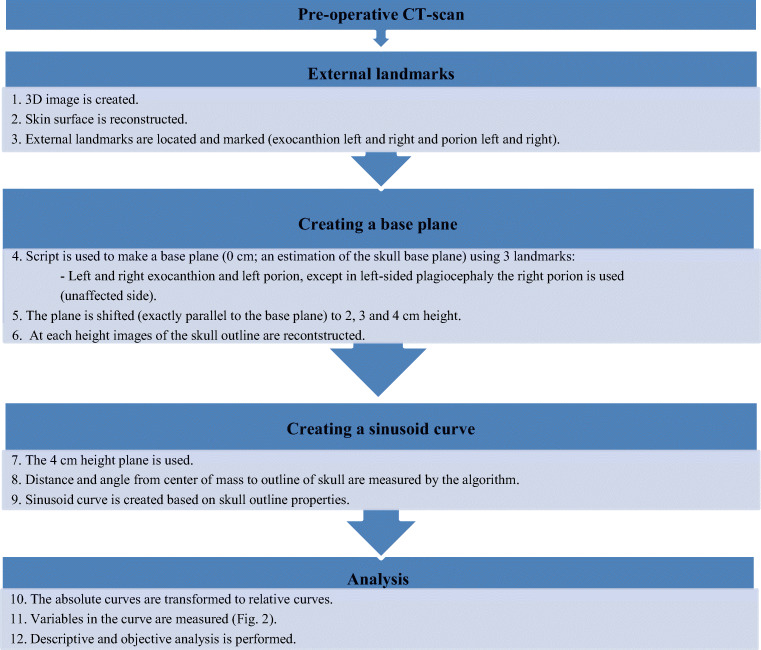
Summary of methods

In order to create sinusoid curves, we used the described methodology of our previous study; a summary of the methods can be found in Fig. 1 [7].

### Analysis

Table 1 shows the extracted and calculated variables from the curves; these values are used for analysis of the curves for each type of craniosynostosis and the control patients.

**Table 1:** Extracted and calculated variables from curve

Extracted variable	Abbreviation	Extracted variable	Abbreviation
Maximum value of forehead peak	F	x-value (in degrees) of the maximum forehead value	XF
Minimum value of left side of head (trough)	L	Minimum value of right side of head (trough)	R
x-value (in degrees) for maximum forehead minus 0.1 (F-0.1) on left side	XFL0.1	x-value (in degrees) for maximum forehead minus 0.1 (F-0.1) on right side	XFR0.1
x-value (in degrees) for maximum forehead minus 0.05 (F-0.05) on left side	XFL0.05	x-value (in degrees) for maximum forehead minus 0.05 (F-0.05) on right side	XFR0.05
x-value (in degrees) of minimum value of width on left side	XL	x-value (in degrees) of minimum value of width on right side	XR
Calculated variable	Formula	Calculated variable	Formula
Width of frontal peak ratio	(XFL0.1-XFR0.1)/(F-0.1)	Asymmetry ratio of frontal peak	(XF-XR)/(XL-XF)
Width of frontal peak at F-0.05	XFL0.05 - XFR0.05	Vertical rise (ΔY)	F-R and/or F-L
Horizontal run (ΔX)	XF-XR and/or XL-XF	Gradient	ΔY/ΔX
Ratio of gradient affected to unaffected leg of curve (in UCS)	Gradient affected side/gradient unaffected side	Ratio of gradient right to left leg of curve (all groups, except UCS)	Gradient right side/gradient left side

For each type of skull shape, the mean, minimum, and maximum values were established for extracted and calculated variables.

**Fig. 2:**
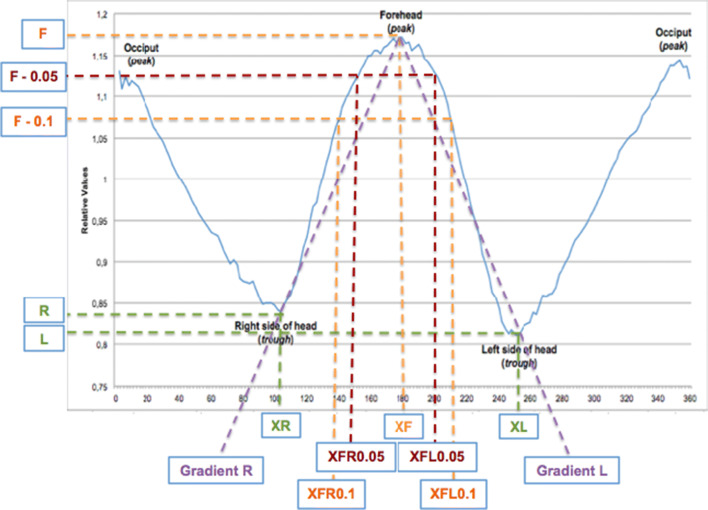
Resulting sinusoid curve; the different variables are marked

With the aim of developing a diagnostic flowchart, we reviewed variables of the 25 included patients that we used to establish diagnostic rules. We listed the known and in literature described clinical appearances of each included subgroup of craniosynostosis: scaphocephaly has a long and narrow skull; brachycephaly has a broad and short skull; trigonocephaly has a long, pointy forehead; and left- and right-sided UCS have an asymmetrical forehead [7]. In our previous study, we showed that these typical clinical findings in subgroups of craniosynostosis can be expressed quantifiably by extracting specific characteristics [7]. Based on the clinical features, we extracted the related specific characteristics of the created sinusoid curves of each simple craniosynostosis type (Table 1; Fig. 2). We reviewed which values and variables were distinctive for the diagnosis and did not show overlap with other subgroups. Cut-off values were based on comparison with the subgroups in which the feature was not distinctive. Based on these rules, we developed a flowchart representing clinical appearance.

The established flowchart was validated by using the (described) variables for the independent validation cohort of 51 patients. The data of these patients were run through the flowchart.

## Results

### Demographics

For validation, we additionally included 51 patients: 16 scaphocephaly patients, 10 brachycephaly patients, 14 trigonocephaly patients, and 6 left-sided and 5 right-sided UCS patients.

Tables 2 and 3 show the demographics of both the initially included patients (*N* = 25) and the included patient for validation.

**Table 2:** Patient characteristics, extracted and calculated variables of initially included patients

	Controls (*n* = 5)	Scaphocephaly (*n* = 5)	Brachycephaly (*n* = 5)	Trigonocephaly (*n* = 5)	Left-sided UCS (*n* = 5)	Right-sided UCS (*n* = 5)
Age (months) (mean (min.–max.))	49 (37–66)	5 (2–9)	4 (2–5)	5 (1–8)	7 (2–11)	9 (3–18)
Sex (male vs. female)	5 vs. 0	5 vs. 0	1 vs. 4	3 vs. 2	0 vs. 5	1 vs. 4
F (mean (min.–max.))	1.13 (1.11–1.17)	1.16 (1.10–1.20)	1.08 (1.05–1.12)	1.18 (1.13–1.22)	1.11 (1.06–1.14)	1.11 (1.07–1.14)
XF (mean (min.–max.))	181 (171–192)	177 (170–184)	174 (172–178)	182 (176–190)	158 (150–164)	202 (194–212)
L (mean (min.–max.))	0.87 (0.85–0.89)	0.81 (0.78–0.85)	0.93 (0.91–0.95)	0.89 (0.84–0.92)	0.92 (0.87–0.95)	0.90 (0.86–0.93)
R (mean (min.–max.))	0.87 (0.81–0.97)	0.81 (0.77–0.85)	0.92 (0.87–0.97)	0.92 (0.89–0.95)	0.90 (0.88–0.96)	0.91 (0.87–0.94)
XFL0.1 (mean (min.–max.))	223 (212–230)	221 (216–228)	228 (196–156)	205 (196–212)	218 (186–274)	229 (218–236)
XFR0.1 (mean (min.–max.))	138 (124–160)	129 (118–138)	108 (96–144)	154 (144–158)	122 (116–130)	145 (128–180)
XFL0.05 (mean (min.–max.))	213 (202–218)	213 (202–218)	227 (182–276)	196 (190–200)	187 (174–210)	220 (212–224)
XFR0.05 (mean (min.–max.))	165 (148–178)	147 (130–170)	118 (106–140)	164 (156–170)	134 (122–140)	171 (152–186)
XL (mean (min.–max.))	265 (248–298)	267 (254–280)	282 (254–338)	250 (236–272)	281 (270–292)	264 (250–284)
XR (mean (min.–max.))	102 (90–114)	85 (72–94)	80 (54–98)	84 (74–102)	90 (78–98)	94 (58–126)
Width of frontal peak ratio (mean (min.–max.))	79 (65–93)	95 (60–167)	123 (69–169)	48 (34–66)	96 (60–173)	83 (50–132)
Width of frontal peak at F-0.05 (mean (min.–max.))	66 (48–80)	72 (54–88)	108 (70–162)	32 (24–40)	54 (40–76)	48 (38–66)
Asymmetry ratio of frontal peak (mean (min.–max.))	1.1 (0.9–1.3)	1.0 (1.0–1.1)	1.1 (0.9–1.2)	1.4 (1.3–1.5)	0.6 (0.4–0.7)	1.8 (1.3–2.4)
Ratio of gradient (slope) (mean (min.–max.))	0.9 (0.5–1.5)	1.3 (0.7–2.0)	1.1 (0.7–1.6)	0.7 (0.5–0.8)	0.5 (0.3–0.8)	0.7 (0.4–0.9)

Green is high(est) value, and red is low(est) value characteristic for skull shape/diagnosis

**Table 3:** Patient characteristics, extracted and calculated variables of patients of the validation set

	Scaphocephaly (*n* = 16)	Brachycephaly (*n* = 10)	Trigonocephaly (*n* = 14)	Left-sided UCS (*n* = 6)	Right-sided UCS (*n* = 5)
Age (months) (mean (min.–max.))	6 (1–11)	6 (1–12)	7 (1–12)	6 (1–11)	7 (1–12)
Sex (male vs. female)	13 vs. 3	5 vs. 5	12 vs. 2	5 vs. 1	2 vs. 3
F (mean (min.–max.))	1.19 (1.14–1.24)	1.05 (1.03–1.09)	1.15 (1.10–1.20)	1.07 (1.07–1.15)	1.07 (1.03–1.10)
XF (mean (min.–max.))	178 (148–204)	136 (104–186)	182 (176–186)	153 (138–168)	195 (162–208)
L (mean (min.–max.))	0.78 (0.71–0.83)	0.95 (0.91–0.98)	0.89 (0.82–0.92)	0.90 (0.86–0.94)	0.91 (0.87–0.93)
R (mean (min.–max.))	0.79 (0.71–0.84)	0.94 (0.89–0.98)	0.91 (0.86–0.94)	0.90 (0.86–0.97)	0.93 (0.84–0.97)
XFL0.1 (mean (min.–max.))	219 (206–232)	229 (178–288)	208 (198–218)	209 (184–232)	230 (218–246)
XFR0.1 (mean (min.–max.))	133 (104–148)	81 (58–110)	150 (120–170)	117 (96–138)	117 (104–132)
XFL0.05 (mean (min.–max.))	210 (198–218)	199 (142–260)	197 (190–206)	189 (170–224)	212 (178–226)
XFR0.05 (mean (min.–max.))	145 (122–158)	107 (88–146)	165 (148–178)	132 (112–150)	159 (124–176)
XL (mean (min.–max.))	268 (250–282)	270 (208–288)	244 (224–262)	244 (206–268)	253 (246–264)
XR (mean (min.–max.))	86 (72–98)	69 (34–90)	94 (36–126)	92 (74–116)	92 (58–154)
Width of frontal peak ratio (mean (min.–max.))	79 (62–106	151 (69–232)	56 (35–90)	93 (63–124)	109 (95–117)
Width of frontal peak at F-0.05 (mean (min.–max.))	65 (46–76)	93 (28–166)	32 (22–44)	57 (46–88)	53 (48–60)
Asymmetry ratio of frontal peak (mean (min.–max.))	1.0 (0.6–1.5)	0.6 (0.3–1.0)	1.6 (0.9–3.0)	0.7 (0.5–0.8)	1.9 (1.2–2.5)
Ratio of gradient (slope) (mean (min.–max.))	1.0 (0.6–1.6)	2.7 (1.1–2.7)	0.6 (0.3–1.0)	0.7 (0.4–0.9)	0.4 (0.3–0.5)

### Extracted and calculated variables

Sinusoid curves are initially made for the 5 patients of each subgroup. The extracted and calculated variables are presented in Table 2. Characteristic features are marked. Additionally, Table 3 shows the variables of the patients included for validation.

### Flowchart

**Fig. 3:**
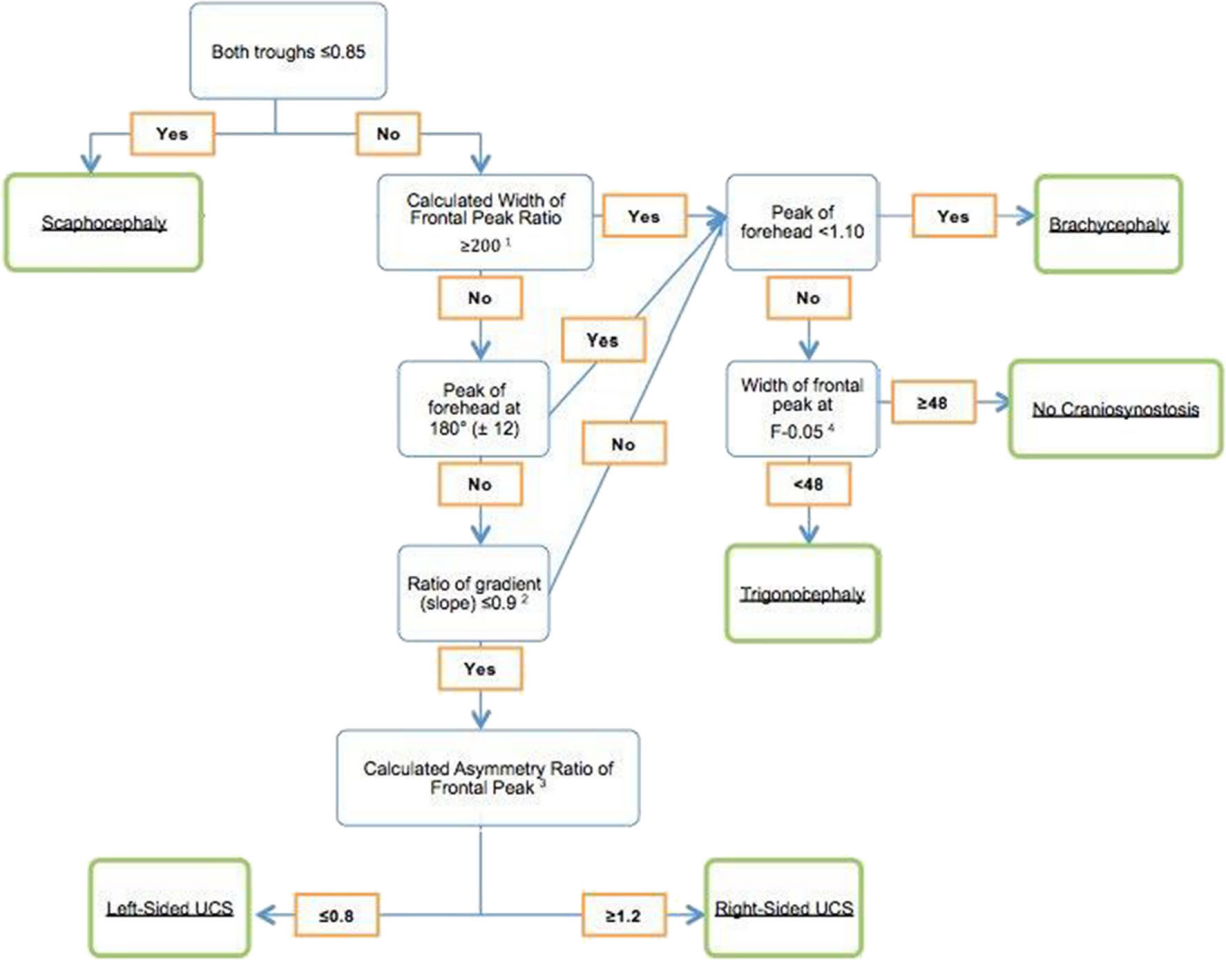
Approach to diagnosis of craniosynostosis. For examples, see Fig. 2. ^1^ Width of frontal peak ratio: (XFL0.1 – XFR0.1) / (F – 0.1). ^2^ Ratio of gradient (slope): gradient affected side/gradient unaffected side or gradient right side/gradient left side. ^3^ Asymmetry ratio of frontal peak: (XF – XR)/(XL – XF). ^4^ Width of frontal peak at F-0.05: (XFL0.05 - XFR0.05)

Figure 3 shows the diagnostic flowchart. A width of frontal peak ratio of ≥ 200 was used to describe a broad peak. We determined if the peak of forehead is at 180° ± 12°. This value of 12° corresponds to the used value of > 3.5% in the CVAI (cranial vault asymmetry index) (3.5% of 360° corresponds to a value of 12.6), which shows significantly asymmetrical values of the head in plagiocephaly patients [8]. An asymmetry ratio of ≤ 0.8 was used to describe a peak shifted to the left side between the troughs and ≥ 1.2 for a peak shifted to the right side between the troughs. A ratio of 0.8 to 1.2 equals no shifting of the forehead peak. We have developed a decision tree based on the training set of 25 patients putting the previously marked variables into a logical and distinctive order to categorize different types of skull shape deformities or normal skull shape. All included patients run through this flowchart towards the CT-confirmed diagnosis.

When using the proposed flowchart for the 51 patients for validation, each of the patients fall into the right category of diagnosis.

## Discussion

The present study proposes a decisive and descriptive flowchart. This flowchart is based on our previously published objective classification method for craniosynostosis [7]. The current study shows the applicability of the method by generating a flowchart in order to diagnose craniosynostosis.

Every subtype of craniosynostosis, as well as the control group, has specific features. These can be seen on clinical examination and are expressed in number by the created sinusoid curves and the extracted and calculated variables.

The presented flowchart is an important step in the process of understanding and the automation of the curves. Using the distinctive characteristics of each patient group, one comes to the diagnosis of a subtype of craniosynostosis. By using external landmarks, our method may be applicable on all 3D surface-rendering techniques, and therefore in the future, a combination of our method (applied on 3D photogrammetry) and the flowchart may be sufficient to diagnose craniosynostosis without the use of CT scan. Making the craniosynostosis diagnosis based on 3D photogrammetry will bring the advantages of no radiation load and no need for sedation in children.

The extracted and calculated variables from the initial group of 25 synostosis and control patients led to decision-making rules. The rules led to a flowchart, which was validated with the use of an independent set of patients (*N* = 51). The first step in the generated flowchart is to determine the troughs of the frontal peak; it can be seen that scaphocephaly has distinctive low values of both troughs (sides of head). The combination of a high forehead peak and additionally the low troughs comes to expression in a long and narrow skull shape. Brachycephaly patients have a relatively low peak of forehead and higher troughs, indicating a broad skull with no prominent protruding forehead. Additionally, some brachycephaly patients have a distinctive large width of frontal peak ratio (≥ 200). In UCS, the peak of forehead is shifted away from 180° ± 12°, ratio of gradient (slope) (affected to unaffected side) is distinctive (≥ 0.9), and an asymmetry ratio of frontal peak ≤ 0.8 or ≥ 1.2 is found, indicating the skewed (fore)head. The unilateral flattening of a leg of the forehead peak is seen in UCS patients, depending on the side of synostosis and coming to expression in the ratio of gradient. Trigonocephaly patients have a relatively high peak of forehead and troughs and a characteristic narrow frontal peak at F-0.05 (< 48). Control patients have a symmetrical skull shape with a relatively high peak of forehead, lower troughs, and a broader frontal peak. It should be noted that the included control patients were of a different age than the patients with craniosynostosis; this is due to the quality of CT scan in most control patients, the area of interest of the CT scan, and our exclusion criteria. However, by using relative values for the curve, curves are adjusted to age and skull size.

All included patients were run through the flowchart and are categorized according to the right CT-confirmed diagnosis. Our flowchart gives a high probability of a certain diagnosis; in the present study for all included patients for validation (*N* = 51), sensitivity and specificity were both 100%.

Being able to categorize patients according to distinctive features extracted from the curves and subsequently make a (correct) diagnosis based on these features is the first step in quantification of severity of each subgroup of craniosynostosis. These distinctive features are now identified and can be used for quantification of the severity of the different diagnoses. However, further research is needed to make a quantification method for each subgroup of craniosynostosis.

In conclusion, we have established and validated a new approach for the classification of different types of craniosynostosis. Every type of craniosynostosis has a specific and recognizable skull deformity, and therefore, we can identify a trend towards a specific and characteristic pattern of the curve for the different types. Based on the curve and values contributing to the curve, it will be possible to diagnose the specific type of craniosynostosis using a novel diagnostic flowchart. This method and flowchart can be a useful tool in the field of research of craniosynostosis and automated diagnosis and may be in the future applicable to 3D photogrammetry.

## Data Availability

N/A.

## Abbreviations

3D: Three-dimensional
CT: Computed tomography
F: Forehead peak
L: Minimum value of left side of the head
O: Occiput peak
R: Minimum value of right side of the head
UCS: Unilateral coronal synostosis
UCSQ: Utrecht Cranial Shape Quantifier
XF: X-value of maximum forehead value
XFL0.05: X-value for the maximum forehead minus 0.05 on the left side
XFR0.05: X-value for the maximum forehead minus 0.05 on the right side
XFL0.1: X-value for the maximum forehead minus 0.1 on the left side
XFR0.1: X-value for the maximum forehead minus 0.1 on the right side
XL: X-value of the minimum value of the width on the left side
XR: X-value of the minimum value of the width on the right side
